# Kidney Lipidomics by Mass Spectrometry Imaging: A Focus on the Glomerulus

**DOI:** 10.3390/ijms20071623

**Published:** 2019-04-01

**Authors:** Imane Abbas, Manale Noun, David Touboul, Dil Sahali, Alain Brunelle, Mario Ollero

**Affiliations:** 1 Lebanese Atomic Energy Commission, NCSR, Beirut 11-8281, Lebanon; imane.abbas@cnrs.edu.lb (I.A.); Manale.Noun@cnrs.edu.lb (M.N.); 2 Institut de Chimie des Substances Naturelles, CNRS UPR 2301, Univ. Paris-Sud, Université Paris-Saclay, Avenue de la Terrasse, 91198 Gif-sur-Yvette, France; David.TOUBOUL@cnrs.fr; 3 Institut Mondor de Recherche Biomédicale, INSERM, U955 EQ21, 8, rue du Général Sarrail, 94010 Créteil, France; dil.sahali@inserm.fr; 4 Université Paris Est Créteil, 61, avenue du Général de Gaulle, 94010 Créteil, France; 5 Hôpital Henri Mondor, 51 avenue du Maréchal de Lattre de Tassigny, 94010 Créteil, France; 6 Laboratoire d’Archéologie Moléculaire et Structurale, LAMS UMR8220, CNRS, Sorbonne Université, 4 place Jussieu, 75005 Paris, France; Alain.BRUNELLE@cnrs.fr

**Keywords:** MALDI-TOF, TOF-SIMS, glomerulopathies, nephrotic syndrome, MSI

## Abstract

Lipid disorders have been associated with glomerulopathies, a distinct type of renal pathologies, such as nephrotic syndrome. Global analyses targeting kidney lipids in this pathophysiologic context have been extensively performed, but most often regardless of the architectural and functional complexity of the kidney. The new developments in mass spectrometry imaging technologies have opened a promising field in localized lipidomic studies focused on this organ. In this article, we revisit the main works having employed the Matrix Assisted Laser Desorption Ionization Time of Flight (MALDI-TOF) technology, and the few reports on the use of TOF-Secondary Ion Mass Spectrometry (TOF-SIMS). We also present a first analysis of mouse kidney cortex sections by cluster TOF-SIMS. The latter represents a good option for high resolution lipid imaging when frozen unfixed histological samples are available. The advantages and drawbacks of this developing field are discussed.

## 1. Introduction: The Kidney Glomerulus as a Witness and Target of Lipid Disorders

The renal glomerulus is a highly specialized anatomic structure containing the filtration barrier that separates the blood and urine compartments. This barrier comprises a fenestrated endothelium, a basement membrane, and a layer of podocytes, highly differentiated postmitotic epithelial cells. Alterations in this barrier lead to proteinuria, which is one of the hallmarks of nephrotic syndrome (NS), along with hypoalbuminemia, hyperlipidemia, lipiduria, and edema. NS characterizes a group of diseases of either genetic or non-genetic—most likely immune—origin. The latter are known as idiopathic nephrotic syndrome (INS), which comprehends two main histologically-defined forms, namely minimal change nephrotic syndrome (MCNS) and focal and segmental glomerulosclerosis (FSGS). Other glomerulopathies include the immune-mediated membranous nephropathy (MN), IgA nephropathy, C3 glomerulopathies, lupus nephritis, anti-neutrophil circulating antibody-associated vasculitis, and different forms of glomerulonephritis. In addition, a number of diseases, such as diabetes, obesity and cancer, the exposure to infectious agents and toxic molecules, are associated with glomerular dysfunction and secondary NS.

NS has been associated with a defect in lipoprotein metabolism and major changes in lipoprotein profiling and content (reviewed in [1]). These alterations are attributed to impaired chylomicron and very low-density lipoprotein (VLDL) clearance [2,3,4], which is associated, at least in part, with decreased abundance of lipoprotein lipase, hepatic lipase, and glycosylphosphatidylinositol-anchored binding protein 1 (GPI-BP1) in several tissues of animal models of NS. Other alterations contributing to NS dyslipidemia are the high serum levels of angiopoietin-like protein 4 in NS patients. The latter has been suggested to play a direct role in the onset of proteinuria [5]. Other alterations include higher levels of cholesterol and LDL-cholesterol, due to increased synthesis and decreased catabolism of cholesterol in the liver [6], and lower levels of HDL-cholesterol to cholesterol ratio in NS patients [7]. Moreover, mutations in the *APOL1* gene, encoding apolipoprotein L1, a key component of high-density lipoproteins (HDL), have been linked to FSGS susceptibility [8,9]. All these alterations lead to higher risk of cardiovascular disease, and to local functional defects in the kidney, such as nephron loss and lipotoxicity in proximal tubular cells. These observations parallel the report of accumulated cholesterol in glomeruli from FSGS patients [10] and in the renal cortex of diabetic nephropathy models [11]. Globally this suggests the presence of profound alterations in the renal and the glomerular lipidome in these pathologic conditions.

Nevertheless, the glomerulus and the podocyte have been scarcely the target of global lipid analyses [12,13]. In an elegant study, Jin and collaborators used a targeted lipidomic approach to unveil the lipid interactors of soluble vascular endothelial growth factor (VEGF) receptor Flt1 at the podocyte surface [13]. They identified the glycosphingolipid ganglioside M3 species as key interactors, and this binding necessary to the functional integrity of podocyte and glomerular filtration barrier. More recently, in a very different setup, lipidomic analysis of urine from pediatric FSGS patients led to the conclusion that these individual display higher free fatty acids and lysophosphatidylcholine (LPC) levels and lower phosphatidylcholine (PC) with respect to controls, suggesting an increased phospholipase A2 activity associated with the FSGS glomerulus [12].

Several pathologies characterized by lipid disorders associated with glomerular alterations have been described in the literature, such as Thay-Sachs, Gaucher, Niemann-Pick, Sandhoff, Fabry, Myopathy 2, HIV-associated nephropathy, and diabetic nephropathy, among others. These disorders are very often characterized by accumulation of different sphingolipid (SP) species (reviewed in [14]). For example, in Fabry disease, the glycosphingolipids globotriaosylceramide (Gb3) and digalactosylceramide (Ga2) significantly accumulate in several tissues, including the kidney, which has been associated with podocyte dysfunction and proteinuria [15]. Sphingomyelin levels, along with those of cholesterol, are increased in the kidney of Niemann-Pick disease, a genetic disorder due to mutations in *NPC1/NPC2* (encoding intracellular cholesterol transporters) and *SMPD1* (encoding an acid sphingomyelinase) genes, the latter associated with proteinuria [16]. Likewise, in genetic forms of nephrotic syndrome due to mutations in the gene *NPHS1* (encoding nephrin, a protein located at the podocyte slit diaphragm, a raft-like membranous structure), there is distal tubular and parietal glomerular accumulation of disyaloganglioside GD3 [17]. Moreover, post-transplant recurrence of FSGS has been found linked to decreased expression of the acid sphingomyelinase-like phosphodiesterase 3b (SMPDL3b) in podocytes, leading to increased sphingomyelin and decreased ceramide [18]. Strikingly, in many of these pathologies the glycosphingolipid metabolic pathway is altered.

## 2. Lipid Imaging of the Kidney: MALDI-MSI

Analysis of kidney lipids by mass spectrometry imaging (MSI) is a relatively recent approach that has made a still discreet yet significant contribution by accompanying histological evaluation, global multiomic (including lipidomics) studies, and further proteomic analyses by MSI. MSI lipidomics has extensively been performed on rodent material by matrix-assisted laser desorption/ionization (MALDI), the mainstream method for MSI, which is characterized by the addition of an organic chemical matrix on biological specimens. This technology has been used on normal tissue [19,20,21,22,23] and in different pathophysiologic contexts, such as acute renal injury [24], diabetes/obesity [25], renal acidosis [26] polycystic kidney [27], drug-induced nephrotoxicity [28,29], bisphenol toxicity [30], polymyxin toxicity [31], Fabry disease [32,33], and in glomerulopathies, such as diabetic nephropathy [34] and IgA nephropathy [35].

Some of these studies have provided valuable information about the spatial localization of lipids in kidney anatomical structures. One example is the thorough study that was performed to determine the distribution of sulfated glycosphingolipids (sulfatides) by MALDI-MSI in combination with source-decay LC-MS/MS in a mouse model of renal acidosis. The latter consisted of invalidation of ceramide synthase, one of the key enzymes in the glycosphingolipid biosynthetic pathway. The technical approach was able to discern the sulfatides characteristic of cortex, medulla, and papillae, according to their sphingoid bases, C18-phytosphingosine, C18-sphingosine, and C20-sphingosine, respectively. In particular, ceramide synthase deficiency was correlated with a depletion in C18-phytosphingosine in the cortex region [26].

In other cases, although not strictly in the context of lipidomics, MSI has been employed to trace an administered drug and its metabolites [28]. The reported study was performed on frozen and paraffin sections of rabbit kidney, and the targeted small molecule, an inhibitor of c-Met tyrosine kinase. This study was interesting in that samples were formaldehyde-fixed, and two technologies, namely MALDI-MSI and DESI-MSI, were used. The authors examined cortex sections and identified unambiguously crystal deposits containing the molecule and some of its metabolites in tubular areas. This underlines the potential of MSI in this type of studies and also the feasibility of analyzing previously-fixed paraffin material, even considering the ion suppression effects of paraffin and of formaldehyde cross-linking.

### 2.1. MSI Analysis of Human Tissue: Lessons from Renal Cell Carcinoma

Much effort has been made in applying the spatial information provided by MSI techniques to the study of human specimens in the context of kidney cancer, the rationale being the search for differences in lipid composition between normal and tumor tissue, which could be used eventually as diagnostic or prognostic markers. Recently, a MALDI variant consisting of gold nanoparticle-enhanced target-based surface-assisted desorption/ionization (SALDI) has been performed to analyze biopsies of renal cell carcinoma [36]. The study resulted in the identification of two markers of tumor tissue, a sodium adduct of diglyceride DG 38:1 and protonated octadecylamide. Moreover, MALDI-MSI was used in parallel to validate the results obtained by HILIC/ESI-MS when comparing patient tumor material with adjacent normal tissue. By targeting polar lipids, such as gangliosides and sulfoglycosphingolipids, and acidic phospholipids [37], MSI results confirmed the findings by HILIC/ESI-MS, consisting of increased phosphatidylinositols, PI 40:5 and PI 40:4, and gangliosides, GM3 34:1, and GM3 42:2, in carcinoma. In another study comparing normal and tumor tissue, MALDI was coupled to an Orbitrap analyzer. The MALDI–Orbitrap method was initially focused on sulfoglycosphingolipids and allowed the identification of more than 120 molecules. The use of multivariate analyses led to the description of a discriminant signature and this correlated with the result of the parallel MSI analysis. The latter showed two distinct markers, namely phosphatidylethanolamine (PE) 36:4 and sulfodihexosyl-ceramide 42:1, decreased and increased in carcinoma tissue, respectively [38]. Another study compared both types of tissues using two parallel approaches, namely touch-spray ionization and DESI-MSI (desorption electrospray ionization-MSI). Multivariate analysis enlightened a signature of discriminant lipid molecules encompassing ion forms of phosphatidylserine (PS 36:1, PS 38:4) and phosphatidylinositol (PI 38:4) from MSI results, while touch-spray led to discriminant phosphatidylcholine (PC)-derived ions [39].

In these examples, MSI often confirmed the results obtained by a LC-MS setup, but in some cases unveiled new differences between both types of tissues, demonstrating the complementarity of both strategies.

### 2.2. Glomerular Disease: The Spatial Limits

A few of those MSI studies targeting the kidney have attempted to address the lipid composition of glomeruli, but the low resolution (i.e., most often in the 30–70 µm range), which roughly corresponds to the mouse glomerular diameter, limits the observations to the renal cortex, as it is unable to discriminate between glomerular and tubular structures. Nevertheless, some remarkable findings have been reported to date.

One of these reports corresponds to a study on IgA nephropathy, the most prevalent glomerular disease, characterized by deposition of immunoglobulin A on the glomerular mesangium. In a spontaneous mouse model of the disease, the authors used a MALDI source coupled to two tandem analyzers, a quadrupole ion trap, and a time-of-flight [35]. The analysis was able to distinguish the renal cortex and the hilum, marked respectively by phosphatidylcholines (PC 38:6 and PC 40:6) and triglycerides (TAG 52:3 and TAG 54:4). A signature characteristic of the diseased mice was composed of PC O-38:6 and PC O-40:7 (ether forms of PC), an analog of platelet activating factor, and a plasmalogen, respectively. In all cases, the presence of 22:6 (docosahexaenoate, DHA) moieties was surprising, since this fatty acid is not known as particularly abundant in kidney tissue. In addition, some ions were detected as characteristic of the IgA model, *m*/*z* 854.6, 856.6, 880.6, and 882.6, but could not be identified. This was a pioneering study in several ways. On the one side, it is the first reported MSI study focused on a glomerular disease. On the other side, the finding of a relevant molecule containing a 22:6 moiety in the kidney, pointing at a particular function for this fatty acid in this organ that is still remaining to be characterized. This is not an irrelevant issue, as in a chronic kidney disease cohort study, low plasma levels of polyunsaturated fatty acids were found associated with increased renal insufficiency [40]. Moreover, oral administration of n-3 fatty acids (DHA and eicosapentaenoate, EPA) was shown to reduce proteinuria in FSGS patients [41], while the DHA metabolite Resolvin D1 protected podocytes in an adriamycin-induced mouse model of glomerular disease [42].

The latter (adriamycin/doxorubicin injection in rodents) represents one of the few available in vivo models of nephrotic syndrome, as one single injection leads to the development of delayed proteinuria. Hara and collaborators [43] combined doxorubicin injection with hypercholesterolemia, in a mouse invalidated for LDL-receptor. Mice exhibited glomerular injuries resembling FSGS, with podocyte alterations and foam cell infiltration in glomeruli. In this model, the authors found by MALDI-MSI at an 80 µm resolution, two ions at *m*/*z* 518.3 and 543.3, identified as Na^+^ adducts of lysophosphatidylcholines LPC 16:0 and LPC 18:0, some of the main components of oxidized LDL, that were abnormally increased in glomerular regions and colocalizing with oxidized phospholipids. These molecules, when administered in vitro to cultured cells induced the expression of adhesion molecules and cytokines, as well as adhesion and migration of macrophages. MSI in this study contributed to unveiling an interesting lipid peroxidation mechanism associated with this mouse model.

Lysophospholipids were also found increased in a mouse model of diabetic nephropathy, along with PE, gangliosides, and sulfoglycosphingolipids (sulfatides) [34]. Glomerular and tubular lesions accompany proteinuria and happen in a high proportion (about one third) of diabetic individuals. The lipid changes reported in this study were, like in the FSGS model cited above, attributed, at least in part, to increased oxidation. This was proven by the use of pyridoxamine, an inhibitor of oxidative processes, as a control condition. The changes were presented as happening in glomeruli and/or tubuli, in spite of a relatively high resolution (between 10 and 40 µm). Interestingly, ganglioside GM3 forms, *m*/*z* 1151.7 (NeuAc-GM3) and *m*/*z* 1167.7 (NeuGc-GM3), showed a pointed pattern in the kidney, specifically colocalized with glomeruli (identified by PAS staining). NeuGc-GM3, the oxidized form, was found increased in diabetic kidneys. The same pattern was found for sulfoglycolipid SB1a t18:0/22:0, *m*/*z* 1407.8, as compared with its non-oxidized form, as well as glucose-modified PE (Amadori-PE, a plasma marker of diabetes). In all cases the increase was corrected with the pyridoxamine treatment. Modified PE; however, was not specific of glomerular regions. These outstanding results indicate that GM3 and sulfated sphingolipids are particularly abundant in glomeruli, most likely in podocytes, and point at this particular lipid class as a target of glomerular alterations in pathologic conditions.

Modifications in the lipid content of glomeruli in diabetes has been the result of another study, devoted to a MALDI-MSI metabolomic profiling of diabetic mice exposed to high-fat diet. In this case the initial search for nucleotide distribution in tissues ended up in a lipid finding. Thus, along with the finding of an altered ATP/AMP ratio, untargeted analysis revealed an accumulation of sphingomyelin SM d34:1 in the glomeruli of those mice as compared to controls [25]. It was argued that the sphingomyelin accumulation could be responsible for the increased ATP production, due to activation of the glycolytic pathway. In this study, the results in glomeruli were obtained with a relatively high-resolution setting (30 µm). Glomeruli were identified using a phosphatidic acid species (PA 36:1) as a glomerular signature. This represents an interesting strategy to be used in the selection of regions of interest in those MSI studies addressing glomerular lipid composition.

Another condition that leads to glomerular dysfunction is renal toxicity due to bisphenol exposure. Experimentally, bisphenol targets glomeruli inducing necrosis, in addition to other lesions, such as cloudy swelling of medulla and interstitial collapsing of renal pelvis, in mice exposed to different concentrations of the compound [30]. The modifications in the cortex were associated with differential distribution of seven lipids, including accumulation of SM d22:0/20:4, TAG 16:0/14:0/16:0, PS 18:0/22:6, and PG 16:0/16:0 and a decrease in DAG (18:0/22:6), PE (20:1/20:4), and PI (16:1/18:1) when compared with control mice. The results suggest that the renal cortex is the most sensitive area to the toxic, and a 2D and 3D model construction coupled with a multivariate analysis pointed to a signature specific of bisphenol exposure, based on increased SM d22:0/20:4 and Cer d18:2/24:1, which could eventually be considered as a marker of toxicity.

Finally, a matrix-free variant of MALDI, such as NALDI-MSI has been used to target renal lipids in normal mouse kidney [44], showing increased presence of a K^+^ adduct of PC 32:0 in the renal cortex as compared to the medulla [45]. NALDI technology, in this case consisting of a Fourier transform ion cyclotron resonance mass spectrometer, was applied in parallel to study the brain and kidney, and compared with MALDI. The authors claimed NALDI to improve mass accuracy and detection efficiency. It could represent an alternative to conventional MALDI-MSI in kidney-based studies.

## 3. The TOF-SIMS Alternative

Imaging by cluster time-of-flight secondary ion mass spectrometry (TOF-SIMS) represents a powerful tool for localized lipidomics. SIMS was first developed in the 1960s and 1970s for surface analysis [46,47]. This technology consists of the bombardment of the sample by a beam of polyatomic ions, which induces desorption/ionization of secondary ions from the sample surface. Polyatomic ion focused beams have been used successfully in the analysis of organic surfaces [48,49,50,51,52,53]. The current development of the technology allows a high lateral resolution of some hundreds of nanometers, which makes the technology particularly well fitted for the analysis of tissue sections [48,52,53,54,55,56,57,58,59,60,61,62]. In addition, no matrix is required for ion generation, which avoids all the drawbacks associated with matrix deposition on tissue.

The analysis of kidney tissue by TOF-SIMS was first performed in rats by gold particle bombardment on silver-coated sections [63,64,65]. In their first report, the authors found a two-fold improvement in sensitivity by silver-coating and attained a 200 nm lateral resolution. They reported a clear patchy distribution of silver-cationized cholesterol ions (*m*/*z* 493.4 and *m*/*z* 495.4). These cholesterol-rich areas were identified as nuclear regions of distal tubular epithelial cells [64]. In another report they attributed high cholesterol levels to glomerular areas [63]. In their third work, Na^+^ ions were found increased in glomerular areas, while phosphocholine (m/z 184) was uniformly localized, along with K^+^ ions.

The only report so far of TOF-SIMS analysis of human kidney biopsies was performed by our group in the context of Fabry disease [66], an X-linked disorder characterized by accumulation of Gb3 and Ga2 in the renal cortex due to a deficiency in α-galactosidase A. Our approach was able to detect both sphingolipid molecules characteristic of this pathology in human material [67]. More recently, a MALDI-MSI approach has detected the same defect in the kidney of α-galactosidase A knockout mice [33]. Apart from these pioneering studies, no systematic analysis of glomerular lipids has been addressed by TOF-SIMS imaging.

## 4. Cluster-TOF-SIMS Analysis of Mouse Kidney Cortex

Here we report the use of cluster-TOF-SIMS to analyze the kidney cortex of normal mice. Three-month old C57B6 mice were sacrificed, kidneys were harvested, embedded in optimal cutting temperature (OCT), and immersed in frozen in liquid nitrogen at −195 °C. Samples were stored at −80 °C. Before analysis, 14 µm-thick tissue sections were obtained at −20 °C using a cryostat (CM3050–S Cryostat LEICA Microsystems, SAS, Nanterre, France), then deposited onto a conductive indium thin oxide (ITO) slide and dried for 15 min under a pressure of a few hectopascals.

We used a TOF-SIMS IV mass spectrometer (ION-TOF GmbH, Münster, Germany) with bismuth ion source and a reflectron time of flight analyzer. Bi_3_^+^ cluster primary ions hit the surface of the tissue section with a kinetic energy of 25 keV and an incidence angle of 45°. The focusing mode which is used are the so-called “high current bunched” mode. Secondary ions are extracted with an energy of 2 keV and are post-accelerated to 10 keV just before hitting the detector. A low-energy electron flood gun is used to neutralize the surface during the experiments. For each sample, two areas of 500 × 500 μm with 256 × 256 pixels were analyzed with a primary ion dose of 2 × 10^12^ ions/cm^2^. The stability of the ion emission of different components is controlled along the analysis, most of ions kept the same intensity during the analysis, so that the analysis can be considered as being done below the so-called static limit. An internal mass calibration has been made with H^+^, H_2_^+^, H_3_^+^, CH_3_^+^, and C_29_H_50_O_2_^+^ ([Vitamin E]^+ •^) ions in the positive ion mode and with H^−^, C^−^, CH^−^, C_2_^−^, C_2_H^−^, C_3_^−^, C_3_H^−^, C_4_^−^, C_4_H^−^, and C_29_H_49_O_2_^−^ ([Vitamin E-H]^−^) ions in the negative ion mode. On each ion image, “MC” represents the maximal number of counts in a pixel and “TC” the total number of counts. The color scales correspond to the interval [0, MC].

The global spectra of glomerular and non-glomerular areas were compared. A total of 136 distinct ions (67 in the positive ion mode and 69 in the negative ion mode) were detected and attributed with one or more potential identities by comparison with the *m/z* values of bona fide standards and/or compared with standards in the ION-TOF database (https://www.iontof.com). Some of these standards have been analyzed in our laboratory and the spectra published [51,57,60,68]. Examples of standard spectra are shown in Figure S1. The *m/z* and attributed identities are presented in Supplementary Tables S1 and S2. Among these ions, 128 were identified as lipids, lipid adducts or lipid fragments, belonging to six different lipid categories (according to Lipid MAPS classification). All of them were detected both in glomerular and non-glomerular areas.

**Figure 1:**
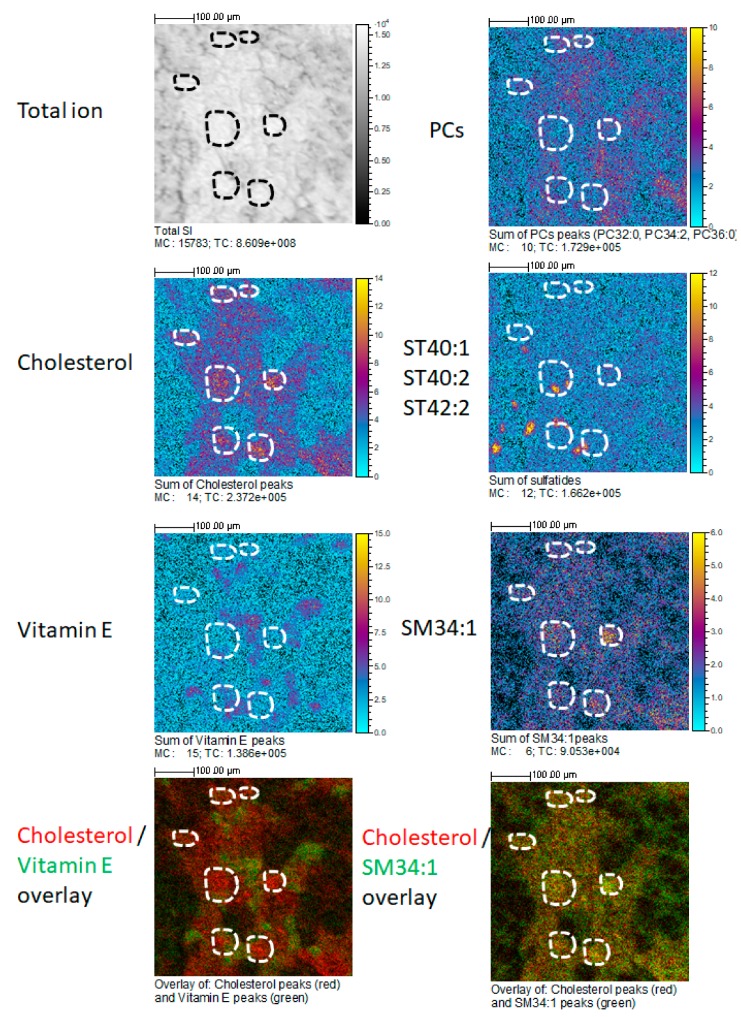
Representative section of a mouse kidney cortex, showing the spatial distribution of several ions (sum of cholesterol peaks, sum of α-tocopherol peaks, sum of phosphatidylcholine (PC) peaks, sum of sulfatides (ST 40:1, ST 40:2, ST 40:3), sum of sphingomyelin (SM) 34:1 peaks, and the total ion current) at a spatial resolution of 2 µm per pixel. The scale on the right (gray levels or colors) indicates the relative abundance. Yellow or white denote higher abundances, while blue or black denote lower abundances. The two lower panels have been constructed by attributing one color (red or green) to each compound. Glomerular regions of interest are selected and depicted by the dashed white line. Bar length: 100 µm. The name of the compound, the maximal number of counts in a pixel (MC), and the total number of counts (TC) are written below each image.

**Figure 2:**
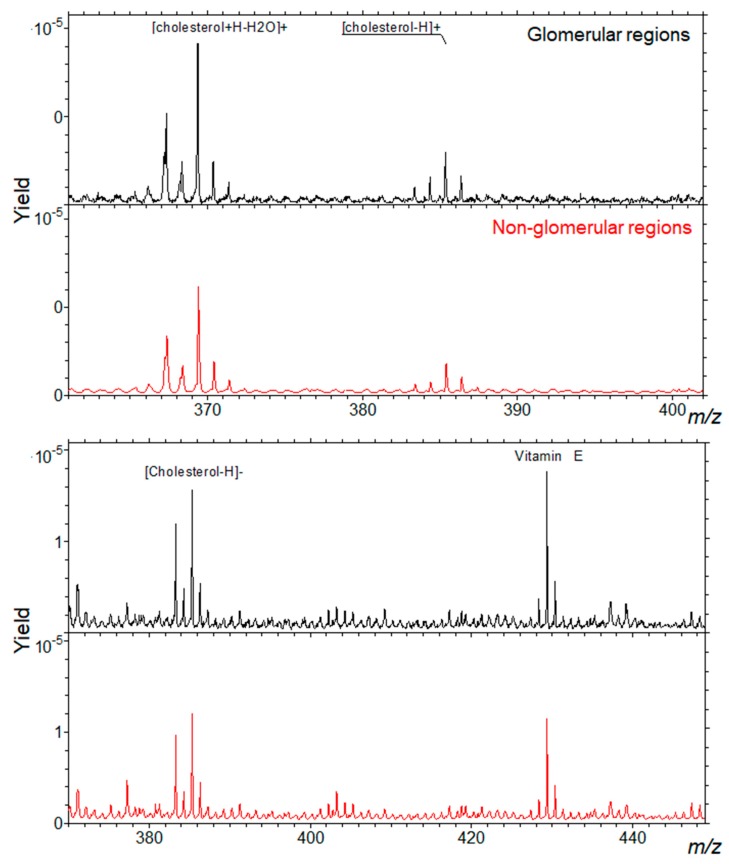
Representative spectra of a mouse kidney cortex, showing the relative intensities of peaks corresponding to cholesterol ions (in positive-top- and negative-bottom-mode) in glomerular and non-glomerular regions. The spectra are normalized to the total primary ion dose.

Figure 1 shows the relative intensity maps of five ions or ion combinations corresponding to the sum of cholesterol peaks, of SM 34:1 peaks, α-tocopherol (vitamin E) peaks, sulfatides ST 40:1, ST 40:2, ST 42:2, and PC 32:0, PC 34:2, PC 36:0. Relative intensity is represented by a color scale, in which yellow corresponds to higher and blue to lower abundances. Glomerular areas were surrounded by a white dashed line. We observed a consistent increased intensity of both cholesterol ions and SM 34:1 in glomerular areas. In the lower panels, one artificial color (red or green) was attributed to each compound, which allowed visualizing the co-localization of either cholesterol and α-tocopherol or cholesterol and SM 34:1. As it can be observed, cholesterol co-localized with SM 34:1 and counter-localized with α-tocopherol (yellowish signal) in all cortical tissue, but intensity was higher in glomerular areas. Sulfatides presented a distinct patchy distribution in non-glomerular regions, while PC ions were distributed more or less homogeneously throughout the renal cortex, but showing a slightly higher abundance (red color) in non-glomerular areas. Figure 2 shows the distribution of cholesterol (in positive and negative mode) in glomerular and non-glomerular regions.

In agreement with our result, a MALDI-MSI-based analysis of rat kidney combined with histological data has reported cholesterol and squalene as specific glomerular metabolites [19]. Cholesterol is; therefore, enriched in glomeruli, but not exclusively. Additionally, we found an enrichment in SM d34:1 in glomerular regions, which could correspond to the same molecule that has been found increased in the glomeruli of a mouse model of diabetic nephropathy in comparison with control animals [25]. Ideally, an exclusive ion could be used for ROI selection in only-glomerular studies. The studies by other groups have proposed interesting glomerular markers, such as PA (36:1). Unfortunately, our TOF-SIMS setup was unable to detect this ion. Glomeruli are, nevertheless, an anatomical structure relatively easy to identify in adjacent sections by conventional staining, and even on ion images. Lipid ions found in glomerular areas can reflect the lipid composition of podocytes, parietal epithelial cells, or glomerular endothelial cells. Our setup can be used to establish modifications in the lipid composition of glomeruli in mouse models of glomerular diseases or in biopsies from patients. Potential changes found in these areas would be complementary to those found in global lipidomic analyses on isolated glomeruli, or on primary podocytes. The setup presented above provides the combination of a good spatial resolution, the unbiased identification of a large number of molecules, and the potential for glomerular ROI selection and subsequent relative quantitation. This setup will be used to explore variations in the localization and intensity of these ions in models of glomerular dysfunction.

## 5. Concluding Remarks: Possible Evolution of Kidney MSI. Application to Glomerulopathies

The advantages of MSI are, mainly, the possibility of a multiplexed analysis of several ions in a single acquisition, and the information about their spatial localization. The drawbacks, the insufficient resolution as compared to immunofluorescence and electron microscopy, and the limited availability of technical platforms, especially TOF-SIMS. Nonetheless kidney MSI has been proven to support and complement other untargeted lipidomic studies.

INS, and in particular MCNS and FSGS, unlike other glomerulopathies, require renal biopsy to establish the right diagnosis. Therefore, these forms are defined histologically, meaning that little information is provided by regular optical microscopy evaluation of tissue sections. MSI could be helpful both in diagnosis, and as an unbiased way of finding molecular mechanisms that could clarify their uncertain pathogenesis. To date, two main technical approaches for MSI of lipids have been used in the context of renal disease. MALDI-TOF and TOF-SIMS cannot be considered as competing approaches, since they can provide complementary information at different levels (Table 1). Firstly, the type of compounds covered by TOF-SIMS are under *m*/*z* 1500, including small molecules, lipids, metabolites, and elements, while the MALSDI-TOF range is over *m*/*z* 200, which includes many lipids along with peptides. TOF-SIMS presents some advantages, like a higher spatial resolution, higher sensitivity for low molecular weight compounds, no need for homogeneous matrix coating, and the potential for 3D mapping. However, it also presents some drawbacks, mainly associated with large fragmentation, often making identification of original molecules problematic. MALDI-TOF is; therefore, more suitable for unambiguous compound identification. In both cases the analysis is relatively long and complex, and always semiquantitative.

New developments in MALDI-MSI aim at increasing spatial resolution. An example is a reported atmospheric-pressure (AP-MALDI) setup applied to brain and kidney imaging, leading to a lateral resolution of 1.4 µm and coupled to an Orbitrap analyzer [69]. This setup has efficiently shown the tissue localization of PC 38:6, PC 40:6, PC 38:1, PC 32:0, PS 40:0, and SM d36:0. On the other side, recent developments in TOF-SIMS aim to give access to a much higher mass resolution, with the recent launch of the Orbi-SIMS instrument [70] to in situ structural analysis of secondary ions through a tandem TOF analyzer (TOF/TOF) [71].

Interestingly, most of the studies published so far on kidney MSI point at alterations of the glycosphingolipid profile in different pathologies involving glomerular dysfunction (Table 2). This lipid class seems to play a significant role in the correct function of the organ, and of the glomerular barrier in particular. In fact, gangliosides are especially abundant at the surface of podocytes [13]. Integrity of the slit diaphragm, a lipid raft-like membranous structure establishing the interaction between foot processes of adjacent podocytes, is a key aspect in filtration barrier function. Sphingolipids (SP) are main components of this structure, and their metabolism seems to play a major role. In our setting, we have found an increased presence of a sphingomyelin species (SM d34:1) in mouse glomeruli. Nevertheless, other relevant lipid classes have been identified throughout MSI lipidomic analyses, such as glycerophospholipids (GP) and glycero-lysophospholipids, and, most importantly, their differential esterifying acyl chains have been unambiguously identified. Glomerular lipidomics is still underexplored, and MSI constitutes a promising strategy to address this point.

## Figures and Tables

**Table 1:** Comparison of the features provided by Time of Flight Secondary Ion Mass Spectrometry (TOF-SIMS) and Matrix Assisted Laser Desorption Ionization - TOF (MALDI-TOF) in the context of mass spectrometry imaging (MSI).

	TOF-SIMS	MALDI-TOF
Analysis	Elemental and molecular analysis	High molecular weight covering a large range of molecules
Compounds	Lipids, glycosphingolipids, cyclopeptides, drugs, metabolites, minerals	Proteins, peptides, lipids, drugs, metabolites
Mass range	*m/z* ≤ 1500	*m/z* > 200
Sample	Dehydrated, no fixation, no matrix	Dehydrated, homogeneous matrix coating
Imaging	Elemental and chemical imaging and mapping in 2D and 3D	Possibility to characterize and visualize in 2D
Spatial resolution	Down to 100–400 nm	5–50 µm
Sensitivity	High sensitivity for trace elements or compounds, in order of ppm to ppb for most species	Low sensitivity for low molecular weight molecules
Overall	Long and complex semi-quantitative analysis	Long and complex semi-quantitative analysis

**Table 2:** Synthesis of reported findings by lipid MSI in renal cortex and glomerular areas. MALDI: matrix-assisted laser desorption ionization. TOF-SIMS: time of flight–secondary ion mass spectrometry. SP: sphingolipids. GP: glycerophospholipids. SL: sterol lipids.

Reference	Species	Technology	Pathology/Condition	Main Finding (ion/molecule)	Lipid MAPS Category	Anatomical Region
[30]	mouse	MALDI	Bisphenol toxicity	SM d22/20:4 Cer d18:2/24:1	SP	Cortex
[35]	mouse	MALDI	IgA nephropathy	PC O-16:0/22:6 PC O-18:1/22:6	GP	Cortex
[34]	mouse	MALDI	Diabetic nephropathy	NeuGc-GM3	SP	Glomeruli
[25]	mouse	MALDI	Diabetic nephropathy	SM d18:0/16:0	SP	Glomeruli
[28]	mouse	MALDI	FSGS (doxorubicin injection model)	LPC 16:0 LPC 18:0	GP	Glomeruli
[33]	mouse	MALDI	Fabry disease model	Gb3; Ga2	SP	Glomeruli
[66]	Human biopsies	TOF-SIMS	Fabry disease	Gb3; Ga2	SP	Glomeruli
[44,45]	mouse	MALDI	normal	PC 32:0	GP	Cortex
[25]	mouse	MALDI	normal	PA 36:1	GP	Glomeruli
[63,64,65]	mouse	TOF-SIMS	normal	Cholesterol	SL	Glomeruli
this report	mouse	TOF-SIMS	normal	Cholesterol SM d34:1	SL-SP	Glomeruli

## Supplementary Materials

Supplementary materials can be found at https://www.mdpi.com/1422-0067/20/7/1623/s1. Table S1: Negative ions detected by TOF-SIMS analysis of mouse renal cortex. Table S2: Positive ions detected by TOF-SIMS analysis of mouse renal cortex. Figure S1: Upper panel, partial positive spectrum of phosphatidylcholine PC32: 0 standard from Sigma. Lower panel, partial negative spectrum of sulfatide 42:2(18:1/24:1) standard from Sigma. The analyses were done at ICSN-CNRS laboratory. Experimental spectra were compared with standard spectra for identity attributions.

## Abbreviations

Cer	Ceramide
DHA	Docosahexaenoate
DESI	Desorption electrospray ionization
GL	Glycerolipid category
GP	Glycerophospholipid category
GPI-BP1	Glycosylphosphatidylinositol-anchored binding protein 1
HILIC	Hydrophilic interaction chromatography
INS	Idiopathic nephrotic syndrome
LC-MS	Liquid chromatography-mass spectrometry
LPC	Lysophosphatidylcholine
MALDI	Matrix-assisted laser desorption ionization
MCNS	Minimal change nephrotic syndrome
MN	Membranous nephropathy
MSI	Mass spectrometry imaging
NALDI	Nanostructure-assisted laser desorption ionization
NS	Nephrotic syndrome
PC	Phosphatidylcholine
PR	Prenol lipid category
SALDI	Surface-assisted laser desorption ionization
SIMS	Secondary ion mass spectrometry
SL	Sterol lipid category
SM	Sphingomyelin
ST	Sulfatide
SP	Sphingolipid category
TOF	Time of flight
VLDL	Very low-density lipoprotein
